# Supplementary figures and images for: Mosaic Segmental and Whole-Chromosome Upd(11)mat in Silver-Russell Syndrome
Source: Genes (Basel). 2021 Apr 16;12(4):581. doi: 10.3390/genes12040581 (PMC8073375; doi:10.3390/genes12040581)

## I-1 of family 1

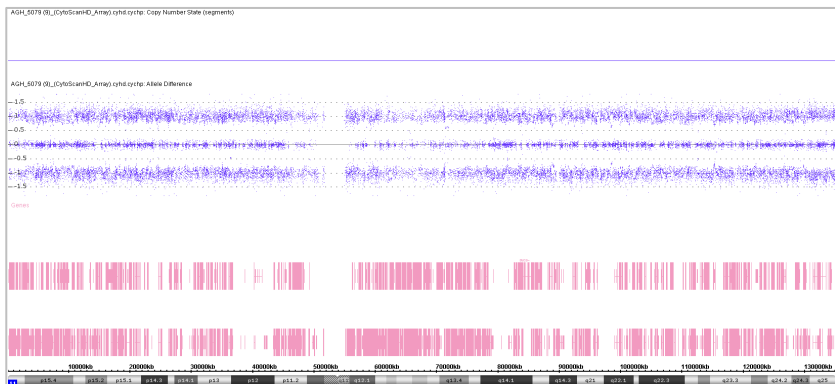

## I-2 of family 1

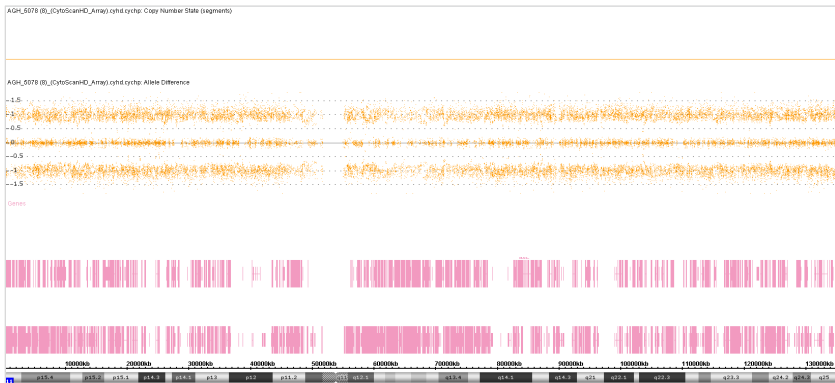

Supplement: Supplementary file 1 [file genes-12-00581-s001.zip › FigureS1.pdf]
